# MiR‐30b‐5p attenuates the inflammatory response and facilitates the functional recovery of spinal cord injury by targeting the NEFL/mTOR pathway

**DOI:** 10.1002/brb3.2788

**Published:** 2022-10-25

**Authors:** Hui Zhang, Duojun Wang, Jinyu Tong, Jianguo Fang, Zaijun Lin

**Affiliations:** ^1^ Department of Spine Surgery Shidong Hospital Affiliated to University of Shanghai for Science and Technology Yangpu District Shanghai China

**Keywords:** inflammation, miR‐30b‐5p, mTOR, neurofilament light chain (NEFL), spinal cord injury (SCI)

## Abstract

**Background:**

Neurofilament light chain (NEFL) has been identified as a biomarker for spinal cord injury (SCI), but its effect and underlying mechanism in SCI remain unclear.

**Methods:**

SCI rat models were established for in vivo studies. Lipopolysaccharide (LPS)‐induced cell models were used for in vitro studies. The protein and mRNA expression levels of genes were evaluated by western blotting and reverse transcription‐quantitative polymerase chain reaction (RT‒qPCR). The pathological changes in rats after SCI were subjected to histological examinations. The interaction of NEFL and upstream miRNAs was explored using dual‐luciferase reporter gene assays.

**Results:**

NEFL was highly expressed in SCI rat spinal cord tissues and LPS‐stimulated PC12 cells. NEFL silencing showed an inhibitory effect on the morphological changes of SCI rats and the secretion of inflammatory factors and facilitated functional recovery of SCI rats. MiR‐30b‐5p was demonstrated to target NEFL and negatively regulate NEFL mRNA and protein levels. Downregulation of miR‐30b‐5p in SCI cell and rat models was demonstrated. MiR‐30b‐5p alleviated the inflammatory response in SCI rat models and LPS‐stimulated PC12 cells and promoted functional recovery in rats by targeting NEFL. NEFL activated mTOR signaling. MiR‐30b‐5p inactivated mTOR signaling by negatively regulating NEFL.

**Conclusion:**

MiR‐30b‐5p alleviated the inflammatory response and facilitated the functional recovery of SCI rats by targeting NEFL to inactivate the mTOR pathway.

## Graphical abstract

Silencing of NEFL inhibits the inflammatory response and functional recovery following spinal cord injury.

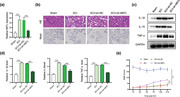

## 1 INTRODUCTION

Spinal cord injury (SCI) is a devastating neurological condition usually caused by external mechanical damage (Alizadeh et al., 2019). Primary and secondary injuries are the two sequential processes of SCI. Patients diagnosed with SCI have poor functional recovery and a high disability rate. During the last three decades, the global SCI cases have increased from 236 to 1238 every million people (Khorasanizadeh et al., 2019), causing a huge burden to patients and society (Badhiwala et al., 2019). Current treatment options for SCI include surgery, glucocorticoids, nerve growth factor, cell therapy, gene targeting therapy, and tissue engineering therapy (Kumamaru et al., 2018; Ozdemir et al., 2012; Song et al., 2019; Sun et al., 2018; Venkatesh et al., 2019). With limited treatment efficiency, it is necessary to explore the underlying mechanism of SCI and find new therapeutic targets.

Neurofilament light chain (NEFL, also named NF‐L) is a neuronal cytoplasmic protein highly expressed in large calibre myelinated axons. The expression of NEFL is elevated in patients with neurological diseases. It is also reported as a potential biomarker for diverse neurological diseases, such as Alzheimer's disease, traumatic brain injury, and frontotemporal dementia (Chouliaras et al., 2022; Gaetani et al., 2019; Shahid et al., 2022). As a sensitive marker for axonal injury, NEFL is also revealed to be highly expressed in patients with motor‐complete SCI and motor‐incomplete SCI and is indicated as a diagnostic and prognostic indicator for SCI treatment (Wang et al., 2022). MicroRNAs (miRNAs) are noncoding single‐stranded RNA molecules approximately 22–24 nucleotides long without protein‐coding potential. MiRNAs are involved in the regulation of gene expression at the posttranscriptional level (Bartel, 2009; Mohr & Mott, 2015). By incorporation into an Argonaute‐containing RNA‐induced silencing complex (RISC), miRNAs bind to the complementary sequences in the 3′ untranslated regions (3′ UTRs) of their target genes (mRNAs) to negatively regulate target gene expression. It has been reported that many miRNAs are dysregulated following central nervous system (CNS) injuries and play critical roles in the inflammatory response and blood‒brain barrier (BBB) disruption of CNS injuries (Pinchi et al., 2019; Sun et al., 2018). The abnormal changes in miRNAs after SCI and their roles in SCI pathological changes have attracted increasing attention. For example, miRNA‐22‐3p expression is decreased in rat spinal cord tissues after ischemia/reperfusion injury, and its upregulation attenuates injury progression by targeting IRF5 (Fang et al., 2021). The upregulation of miR‐324‐5p was observed in spinal cord tissue samples after injury, and its deficiency alleviates the inflammatory response and promotes functional recovery after SCI by modulating Sirt1 (Wang et al., 2021). MiR‐544a inhibits the secretion of inflammatory cytokines and facilitates recovery following SCI by negatively regulating NEUROD4 (Yang et al., 2018).

NEFL is reported to activate the mTOR signaling pathway in the progression of glioblastoma (Peng et al., 2015). mTOR is a serine/threonine protein kinase in the PI3K‐related kinase (PIKK) family and assembles into two complexes, mTORC1 and mTORC2. Previous studies have revealed that mTOR regulates cell growth and metabolism with environmental inputs (Saxton & Sabatini, 2017). mTOR signaling dysfunction contributes to the development of various diseases, including SCI (Kanno et al., 2012). Metformin alleviates SCI by modulating autophagy and reducing apoptosis via inactivation of the mTOR/P70S6K signaling (Guo et al., 2018). Ezetimibe inhibits the activation of PI3K/AKT/mTOR signaling to exert protective effects against SCI (Chen et al., 2020). Netrin‐1 promotes functional recovery after SCI by regulating the AMPK/mTOR pathway (Bai et al., 2017). However, whether NEFL affects the mTOR pathways in SCI progression remains unclear.

In this study, we aimed to investigate the function and underlying mechanism of NEFL in SCI pathology. We hypothesized that NEFL promotes SCI progression by activating mTOR signaling, and its specific regulatory mechanism was explored in vivo and in vitro. The findings of our study may provide insight into the targeted therapy of SCI patients.

## 2 MATERIALS AND METHODS

### 2.1 Animals

This study was approved by the Ethics Committee of Shidong Hospital Affiliated to University of Shanghai for Science and Technology. The experimental procedures were in accordance with the National Institutes of Health Guidelines for the Care and Use of Laboratory Animals. Six‐ to ten‐week‐old male Sprague‒Dawley (SD) rats (230–300 g) were provided by Vital River Laboratory Animal Technology (Beijing, China). The animals were raised at 23–25°C under 12‐h light and dark cycles with free access to food and water.

### 2.2 Establishment of SCI rat models

The rats were randomly divided into seven groups, including sham, SCI, SCI+sh‐NC, SCI+sh‐NEFL, SCI+NC mimics, SCI+miR‐30b‐5p mimics, and SCI+miR‐30b‐5p mimics+NEFL, with 6 rats in each group. The SCI rat models were established as previously described (Zhang et al., 2021). After anesthetizing the rats with pentobarbital sodium (40 mg/kg), with the T10 spinous process as the center, a 2–3 cm incision was made on the midline of the rat back to expose the vertebrae. Then, the T10 spinal cord was exposed by removing the spinous process and lamina. A 2‐N strike force was used to strike the T10 spinal cord. Rats in the sham groups only received laminectomy without being struck. After washing with penicillin saline, the wound was sutured. Rats received antibiotics (gentamicin, 50 mg/kg) and analgesics for the first 3 days after SCI to minimize infection, and the bladders were emptied manually twice daily until recovery of the micturition reflex.

### 2.3 Lentivirus vector construction

sh‐NEFL or sh‐NC (negative control) was ligated into lentiviral vectors (Genechem, Shanghai, China). These lentiviral vectors (1×10^7^/0.05 mL) were then injected into the tail vein of rats 3 days before modeling with a microneedle (Dong et al., 2021). Finally, rats were sacrificed by cervical dislocation on day 21, and spinal cord tissues at the injury epicenter were collected for subsequent analysis.

### 2.4 HE staining

Twenty‐one days after SCI, the rat spinal cords were collected, immersed in xylene, dehydrated in gradient ethanol solutions, and then stained with hematoxylin for 6 min and eosin for 60 s, followed by dehydration through increasing concentrations of ethanol solutions and xylene. The histological changes in rat spinal cord tissues in the indicated groups were observed using an optical microscope.

### 2.5 Nissl staining

Rat spinal cord tissues were obtained from animals in the indicated groups and cut into sections (10 µm thick). Then, 1% cresyl violet (Beyotime, Shanghai, China) was used to stain the tissue sections. The neuron number in each sample was calculated in five randomly chosen visual fields. The calculation of neuron loss was performed with Image‐Pro Plus 6.0.

### 2.6 BBB score

The Basso, Beattie, and Bresnahan (BBB) score was used to evaluate the motor function of rats after injury by three researchers blinded to group treatments. Rat movements in the indicated groups were observed, and rat locomotor function was scored in accordance with the BBB scales (Basso et al., 1995) on days 1, 3, 5, 7, 14, and 21 post modeling. Based on limb movements, coordination, gait, and claw movement, the BBB scores were evaluated and ranged from 0 to 21 points, where 0 indicated no movement, while 21 indicated normal movement. The rats were first detected before modeling to exclude baseline deficits. Finally, the scores of each rat were collected and averaged.

### 2.7 Cell culture and treatment

PC12 cell lines were provided by American Type Culture Collection (ATCC, Manassas, VA, USA). The cells were incubated in DMEM (Thermo Fisher, Shanghai, China) supplemented with 1/100 streptomycin/penicillin and 10% FBS (Thermo Fisher) at 37°C in the presence of 5% CO_2_. LPS (100 ng/ml, Beyotime) was used to stimulate PC12 cells at 37°C for 4 h with 5% CO_2_ to establish SCI cell models (Tan et al., 2018).

### 2.8 Cell transfection

Cells were inoculated into 6‐well plates at 5 × 10^6^ cells/well to reach 80% confluency. Short hairpin RNAs against NEFL (sh‐NEFL), miR‐30b‐5p mimics, and matched negative controls (sh‐NC and NC mimics) were provided by GenePharma (Shanghai, China). The indicated plasmids were transfected into PC12 cells with Lipofectamine 3000 (Life Technologies, MA, USA). The transfected PC12 cells were collected at 48 h for the following study.

### 2.9 Reverse transcription quantitative polymerase chain reaction (RT‒qPCR)

Total RNA was isolated using the TRIzol (Invitrogen) one‐step method. cDNA synthesis was then performed using a first‐strand cDNA synthesis kit (Takara). SYBR Green Master Mix (Thermo Fisher Scientific, USA) was applied for PCR analysis. The reaction conditions were 95°C for 2 min, followed by 40 cycles of denaturation at 95°C for 15 s, annealing at 60°C for 25 s, and extension at 72°C for 1 min. The 2^−ΔΔCt^ method was used for gene expression calculation with GAPDH and U6 as the endogenous controls. The primer sequences are presented as follows: NEFL: F: 5′‐GCCGAAGAGTGGTTCAAGAG‐3′, R: 5′‐TGTCTGCATTCTGCTTGTCC‐3′; IL‐1β: F: 5′‐GGATAACGAGGCTTATGTGCACG‐3′, R: 5′‐GGACATGGAGAACACCACTTGTTG‐3′; IL‐16: F: 5′‐ATTGTATGAACAGCGATGATGCAC‐3′, R: 5′‐CCAGGTAGAAACGGAACTCCAG‐3′; TNF‐α: F: 5′‐ATACACTGGCCCGAGGCAAC‐3′, R: 5′‐CCACATCTCGGATCATGCTTTCA‐3′; miR‐30b‐5p: F: 5′‐CAGTGCAGGGTCCGAGGT‐3′, R: 5′‐AAGCGCCTTGTAAACATCCTACA‐3′; GAPDH: F: 5′‐AACTCCCATTCTTCCACCT‐3′, R: 5′‐TTGTCATACCAGGAAATGAGC‐3′; U6: F: 5′‐CAGTTATGACGACCTAGACAG‐3′, R: 5′‐CAAATTTGCATGTCATCCTTGG‐3′.

### 2.10 Western blot

Total protein was isolated from PC12 cells and rat spinal cord tissues with RIPA buffer (Beyotime). A BCA Protein Assay Kit was applied for protein concentration detection. The proteins were isolated with polyacrylamide gel (Boster) electrophoresis and then loaded onto PVDF membranes. Before overnight culturing with the primary antibodies at 4°C, 5% BSA was applied to block the membranes for 1 h, which were then cocultured with primary antibodies, including NEFL (ab52989, 1:1000, Abcam), IL‐1β (ab254360, 1:1000, Abcam), IL‐16 (PA5‐86635, 1:1000, Thermo Fisher), TNF‐α (PA1‐40281, 1:1000, Thermo Fisher), p70S6K (PA5‐28597, 1:1000, Thermo Fisher) and p‐p70S6K (PA5‐38307, 1:1000, Thermo Fisher) with GAPDH (PA1‐16777, 1:2000, Thermo Fisher) as the internal control. After washing 3 times with Tris‐buffered saline‐Tween 20, the membranes were incubated with the corresponding secondary antibodies (ab205718, 1:2000, Abcam) at room temperature for 1 h. Finally, an enhanced chemiluminescence kit (Thermo Fisher) was used to visualize the protein bands that were analyzed with ImageJ software (NIH, USA).

### 2.11 Luciferase reporter assay

The wild‐type (WT) or mutant (MUT) sequence of the NEFL 3′UTR containing binding sites for miR‐30b‐5p was cloned into the pmiR‐GLO vector (Promega Corporation, Madison, WI, USA). Then, the reporter plasmids were transfected into LC12 cells with miR‐30b‐5p mimics or NC mimics using Lipofectamine 3000 (Invitrogen) for 48 h. The luciferase reporter activities were evaluated using a Dual Luciferase Assay kit (Promega, USA) following the manufacturer's protocol.

### 2.12 Statistical analysis

GraphPad Prism software was used for statistical analysis. The results are expressed as the means ± standard deviations (SD). Student's *t*‐test and one‐way analysis of variance (ANOVA) were applied to assess differences between groups, with a *p* value less than 0.05 considered statistically significant.

## 3 RESULTS

### 3.1 NEFL is upregulated in SCI rat models

**FIGURE 1:**
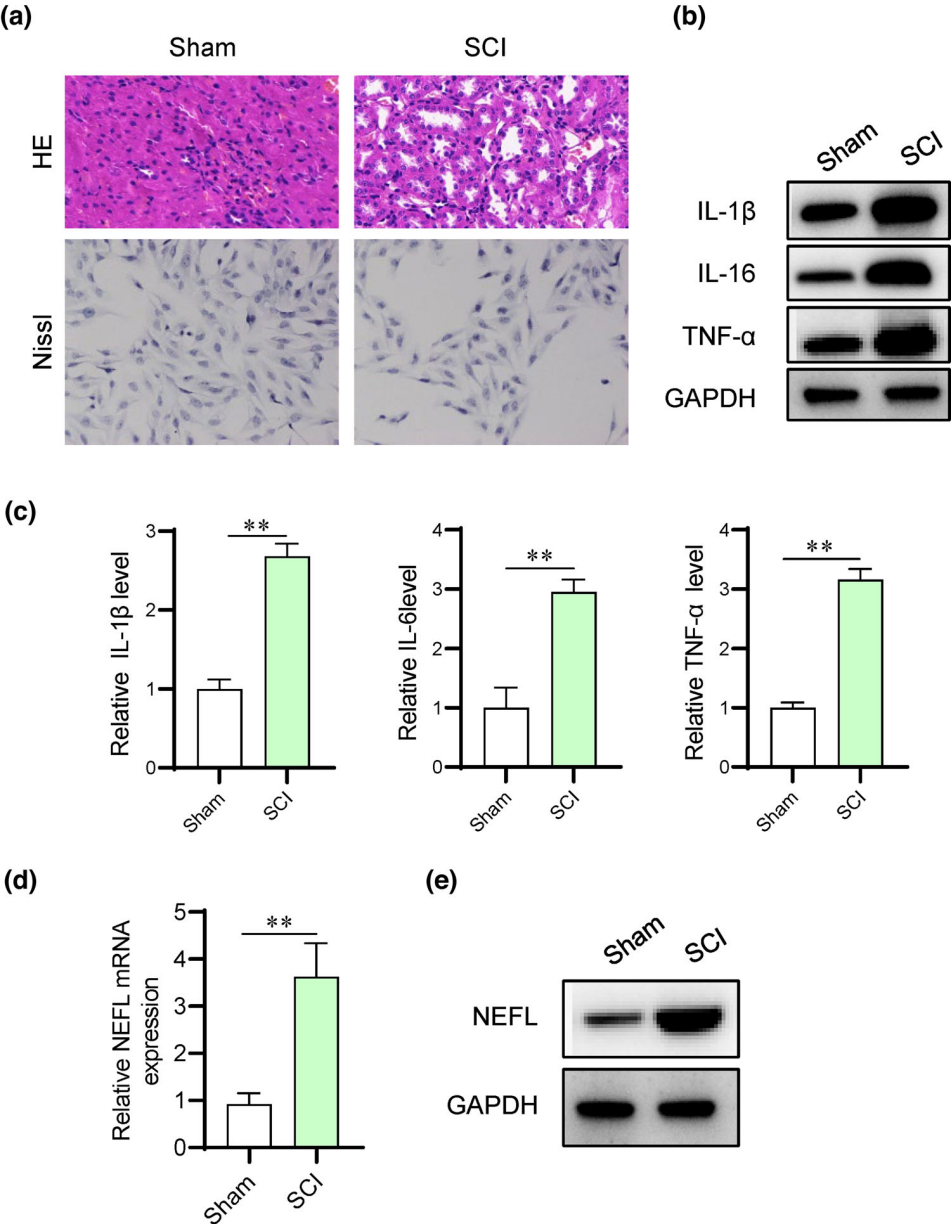
NEFL is upregulated in the spinal tissues of SCI rats. (A) HE staining was performed to detect morphological changes, and Nissl staining was conducted to detect neuronal loss in rat spinal cord tissues. Magnification, 400×. (B‐C) Western blotting was used to measure the protein levels of inflammatory cytokines in the spinal cord tissue samples of rats post spinal cord injury. (D‐E) RT‒qPCR analysis and western blotting were used to detect the mRNA and protein expression of NEFL in the spinal tissues of rats in the indicated groups. **p < 0.01.

To elucidate the biological functions and regulatory mechanism of NEFL in SCI, SCI rat models were established. The pathological conditions of SCI rats were assessed using HE and Nissl staining. As shown in Figure 1, HE and Nissl staining showed that rat spinal tissues underwent severe damage in the SCI group, with an increased cavity area and a reduced number of Nissl bodies in the spinal tissues of SCI rats (Figure 1A). Then, we detected the levels of inflammatory cytokines in the spinal tissues of SCI rats. We observed that IL‐1β, IL‐16, and TNF‐α protein and mRNA expression exhibited significant elevation in the SCI groups in comparison with the sham groups (Figure 1B,C). The mRNA and protein expression of NEFL was demonstrated to be elevated in SCI rat spinal tissues (Figure 1D‐E).

### 3.2 NEFL silencing attenuates the inflammatory response and functional recovery after SCI

**FIGURE 2:**
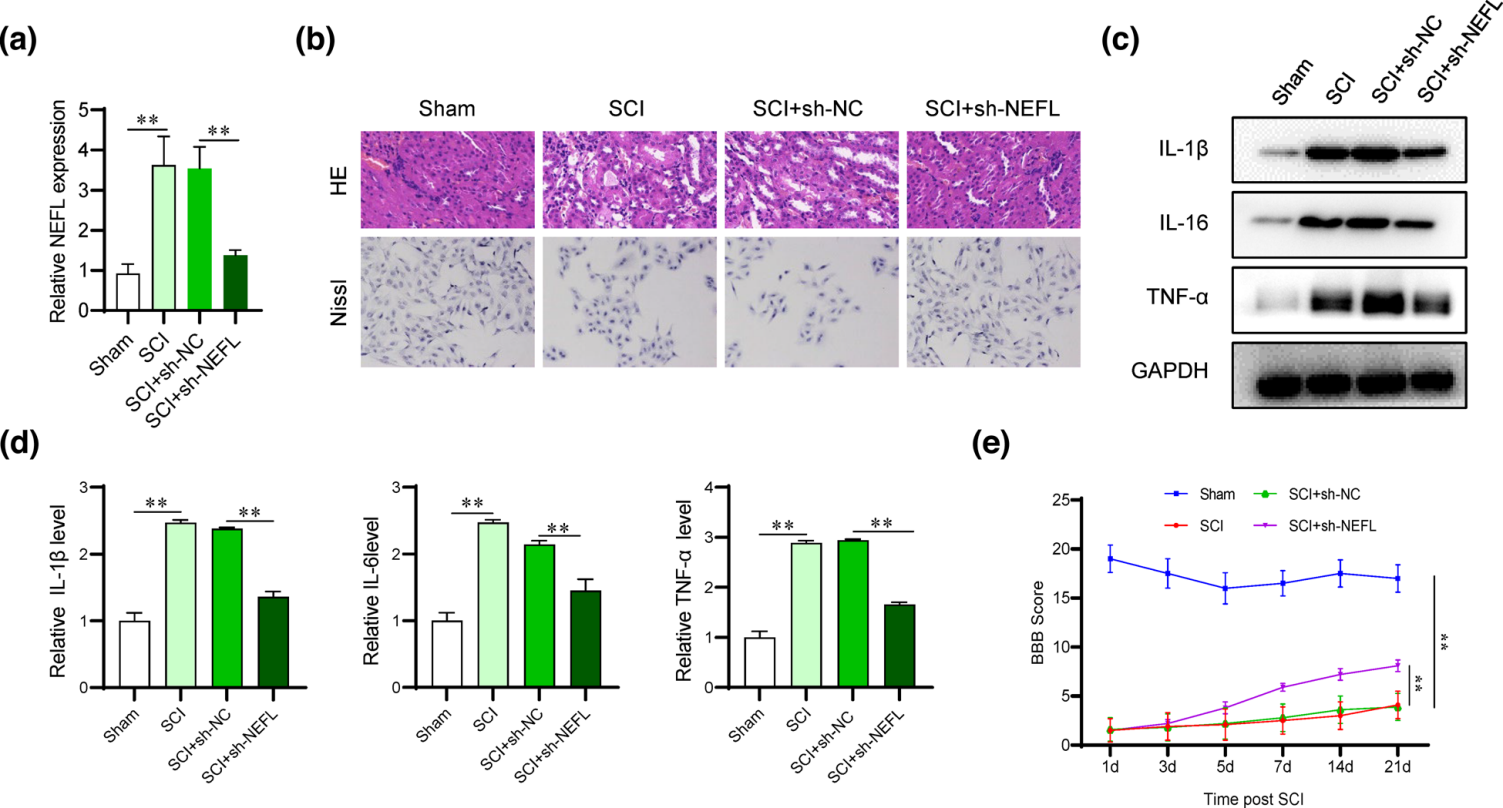
NEFL silencing attenuates the inflammatory response and functional recovery after SCI. (A) The mRNA levels of NEFL in rat spinal cord tissues were subjected to RT‒qPCR analysis. (B) The HE and Nissl staining images for the assessment of spinal cord injury in the indicated groups. Magnification, 400×. (C‐D) Western blot and RT‒qPCR analyses were performed to measure the protein expression and mRNA levels of inflammatory factors in the indicated groups. (E) The BBB motor function scores of rats in the sham, SCI, SCI+sh‐NC, and SCI+sh‐NEFL groups were evaluated and recorded by 3 researchers blinded to group allocation at 1, 3, 5, 7, 14, and 21 days post SCI. **p < 0.01.

We explored the function of NEFL in SCI progression in vivo using loss‐of‐function assays. The silencing efficiency of NEFL was determined using RT‒qPCR analysis. The levels of NEFL were significantly reduced after transfection with sh‐NEFL in SCI rats (Figure 2A). HE and Nissl staining showed that the morphological changes and neuronal loss caused by SCI were alleviated after silencing NEFL (Figure 2B). The SCI‐induced increase in the protein and mRNA expression of inflammatory factors (IL‐1β, IL‐16, and TNF‐α) was reversed by the knockdown of NEFL (Figure 2C‐D). Moreover, we also monitored the BBB score of rats in the sham, SCI, SCI+sh‐NC, and SCI+sh‐NEFL groups 21 d after SCI. The BBB score exhibited a significant reduction in the SCI groups, which was partially rescued after NEFL knockdown (Figure 2E), indicating that NEFL silencing promoted the functional recovery of SCI rats.

### 3.3 NEFL knockdown alleviates LPS‐induced injury in PC12 cells

**FIGURE 3:**
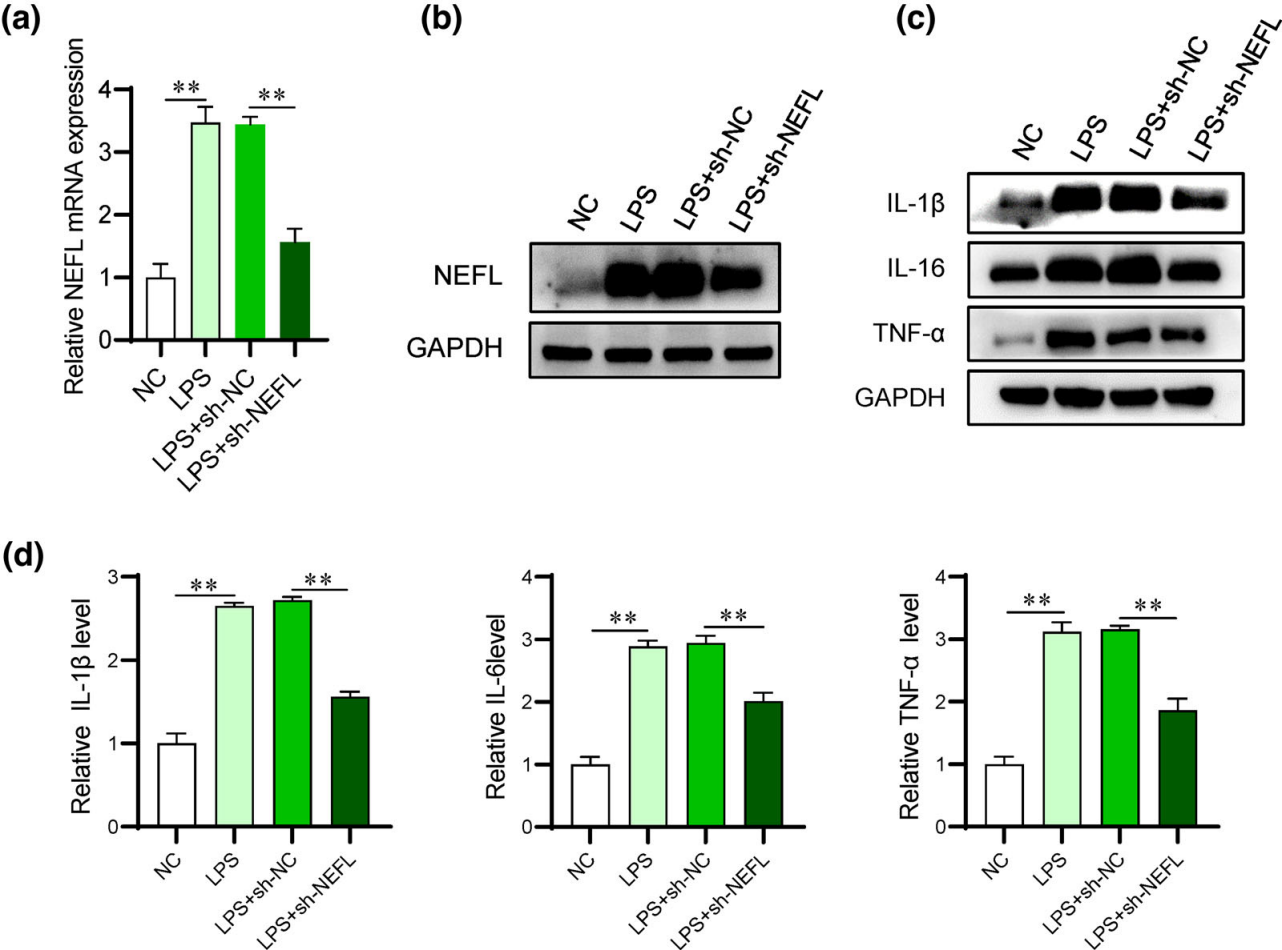
NEFL knockdown alleviates LPS‐induced injury in PC12 cells. (A‐B) RT‒qPCR and western blot analyses were performed to evaluate the mRNA and protein expression of NEFL in LPS‐treated PC12 cells. (C‐D) Western blot and RT‒qPCR analyses were used to detect the protein and mRNA levels of IL‐1β, IL‐16, and TNF‐α in LPS‐treated PC12 cells with the indicated treatments. **p < 0.01.

We established SCI cell models using LPS‐induced PC12 cells to further investigate the effect of NEFL on SCI progression in vitro. The NEFL mRNA and protein levels were upregulated in LPS‐treated PC12 cells and exhibited a significant reduction after transfection with sh‐NEFL (Figure 3A‐B). Moreover, the protein and mRNA expression of inflammatory factors (IL‐1β, IL‐16, and TNF‐α) showed an increase following LPS stimulation in comparison with the NC groups, while NEFL deficiency reversed the upregulation of inflammatory factors caused by LPS in PC12 cells (Figure 3C‐D).

### 3.4 NEFL is targeted by miR‐30b‐5p

**FIGURE 4:**
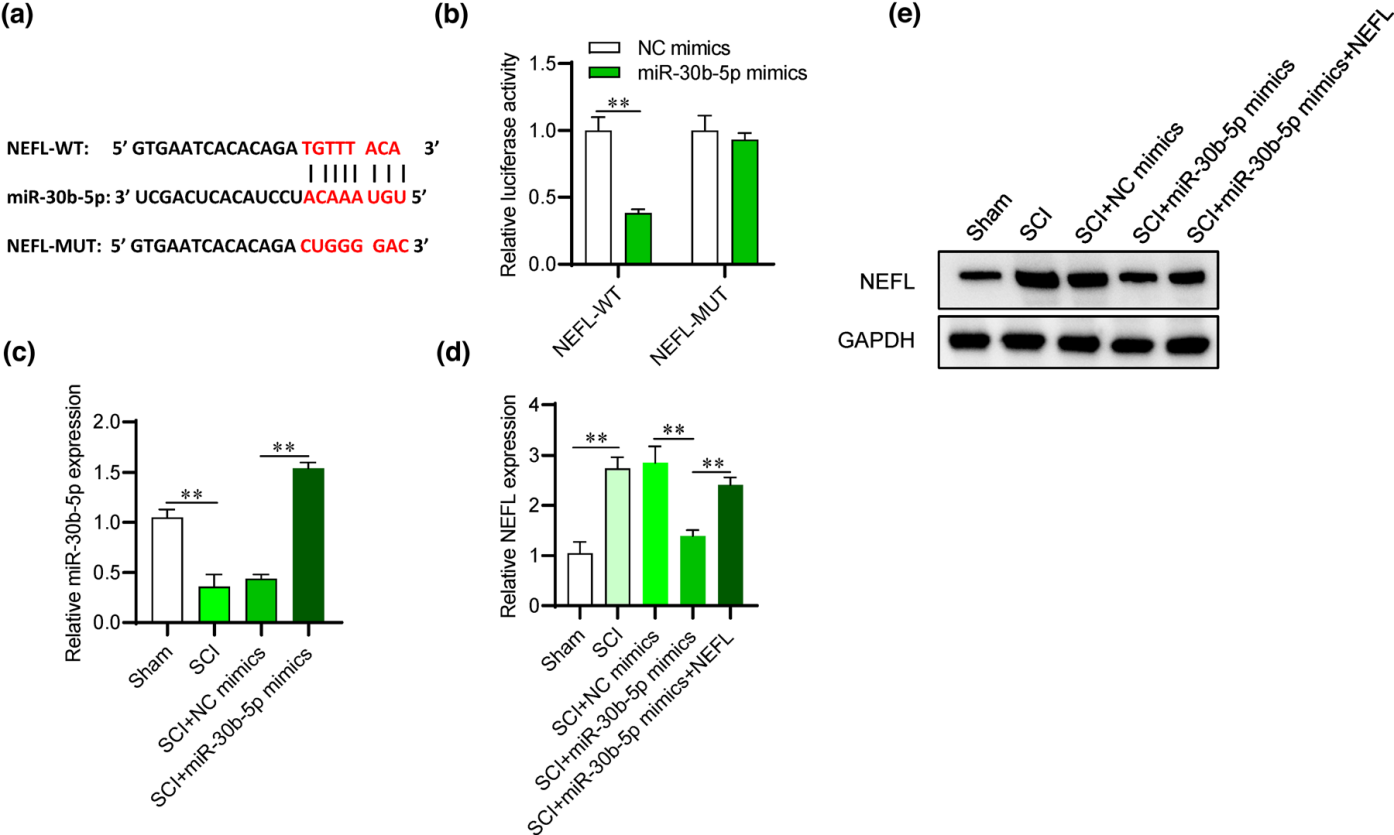
NEFL is targeted by miR‐30b‐5p. (A) The binding sequence of miR‐30b‐5p and NEFL. (B) The interaction between miR‐30b‐5p and NEFL was explored using a luciferase reporter assay in PC12 cells after transfection with NC mimics or miR‐30b‐5p mimics. (C) RT‒qPCR was used to measure the expression of miR‐30b‐5p in spinal cord tissue samples of rats in the indicated groups. (D‐E) RT‒qPCR and western blot analyses were used to detect the mRNA and protein expression of NEFL in rat spinal cord tissues. **p < 0.01.

The underlying regulatory mechanism of NEFL was further explored. Based on the miRDB database (http://mirdb.org/), the first 10 miRNAs on the target rank were selected. Only miR‐30b‐5p and miR‐384‐5p expression exhibited significant reduction in spinal cord tissues of SCI rats. Since the functions of miR‐384‐5p in SCI have been revealed in a previous study (Zhou et al., 2020), miR‐30b‐5p was chosen for the following study. The binding sequence of miR‐30b‐5p and NEFL is shown in Figure 4A. According to the results of the luciferase reporter assay, the relative luciferase activity of PC12 cells with NEFL‐WT showed a reduction after the transfection of miR‐30b‐5p mimics compared with those with NEFL‐MUT (Figure 4B), indicating the interaction of miR‐30b‐5p and NEFL in PC12 cells. The expression of miR‐30b‐5p showed a significant decrease in the SCI rat models, and its overexpression efficiency was confirmed (Figure 4C). The impact of overexpressed miR‐30b‐5p on NEFL expression was evaluated, and the findings indicated that NEFL protein and mRNA expression exhibited a decrease after miR‐30b‐5p overexpression in the spinal cord tissues of SCI rats, while the transfection of NEFL overexpression plasmids reversed this trend (Figure 4D‐E), which indicated that miR‐30b‐5p negatively regulated NEFL expression.

### 3.5 MiR‐30b‐5p attenuates the inflammatory response and promotes the functional recovery of SCI rats by targeting NEFL

**FIGURE 5:**
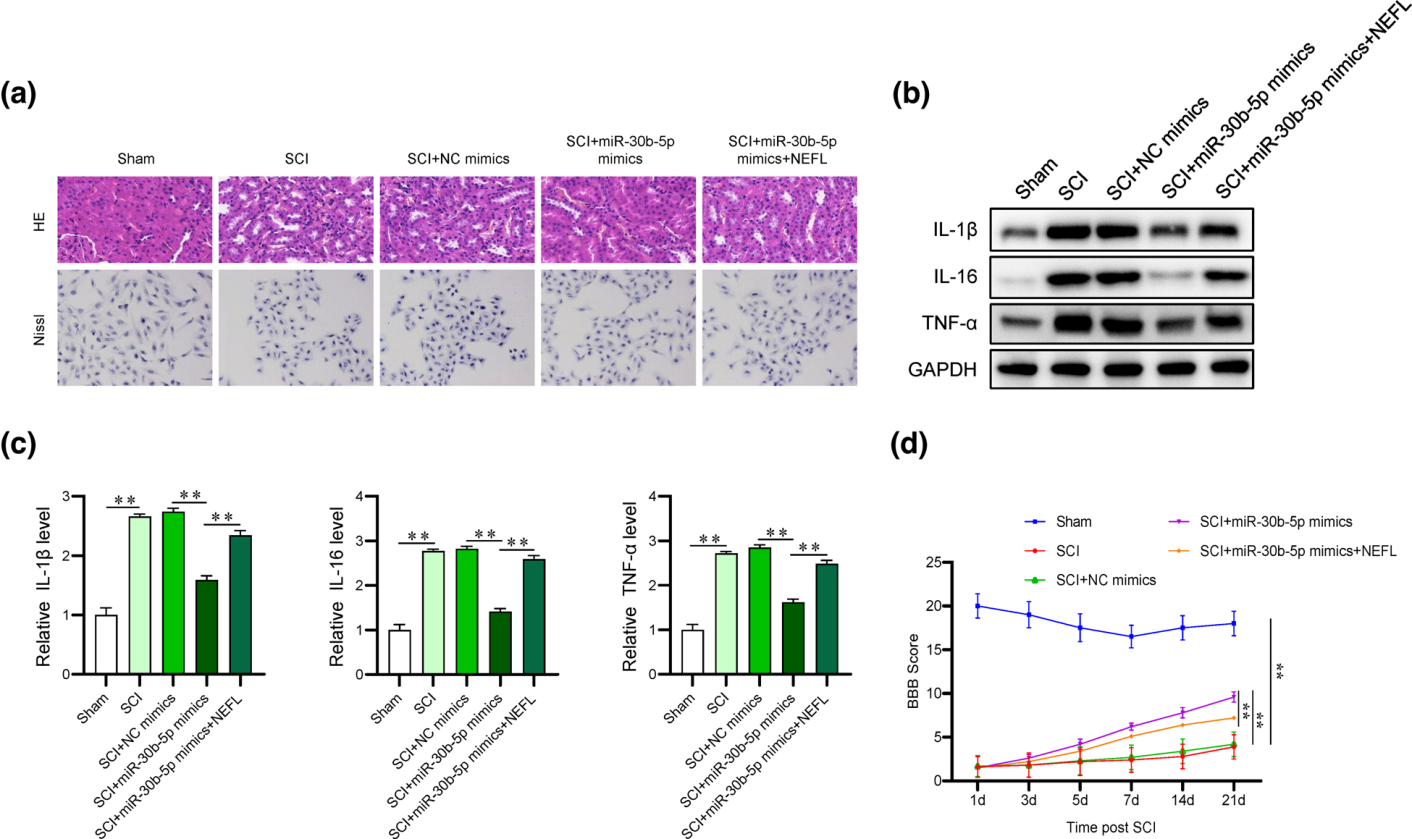
MiR‐30b‐5p attenuates the inflammatory response and facilitates functional recovery after SCI by regulating NEFL. (A) Images of HE and Nissl staining for evaluation of spinal cord injury in the indicated groups. Magnification, 400×. (B‐C) Western blot and RT‒qPCR analyses were used to detect the protein expression and mRNA levels of inflammatory cytokines (IL‐1β, IL‐16, and TNF‐α) in tissue samples of rat spinal cords. (D) The BBB motor function scores of rats at 1, 3, 5, 7, 14, and 21 days post SCI in the indicated groups. **p < 0.01.

We further investigated the effect and underlying mechanism of miR‐30b‐5p on SCI progression in vivo via rescue assays. HE and Nissl staining revealed that miR‐30b‐5p overexpression significantly alleviated the morphological changes and neuronal loss caused by SCI, which was partially reversed after NEFL upregulation (Figure 5A). The elevation in the protein and mRNA levels of inflammatory cytokines (IL‐1β, IL‐16, and TNF‐α) induced by SCI showed a reduction after miR‐30b‐5p upregulation in SCI rat models, while transfection of NEFL rescued the impact exerted by upregulated miR‐30b‐5p on the expression of inflammatory cytokines (Figure 5B‐C). The BBB score indicated that miR‐30b‐5p facilitated functional recovery of SCI rats, which was reversed after transfection of NEFL overexpression vectors (Figure 5D).

### 3.6 MiR‐30b‐5p inhibits the inflammatory response in LPS‐treated PC12 cells by targeting NEFL

**FIGURE 6:**
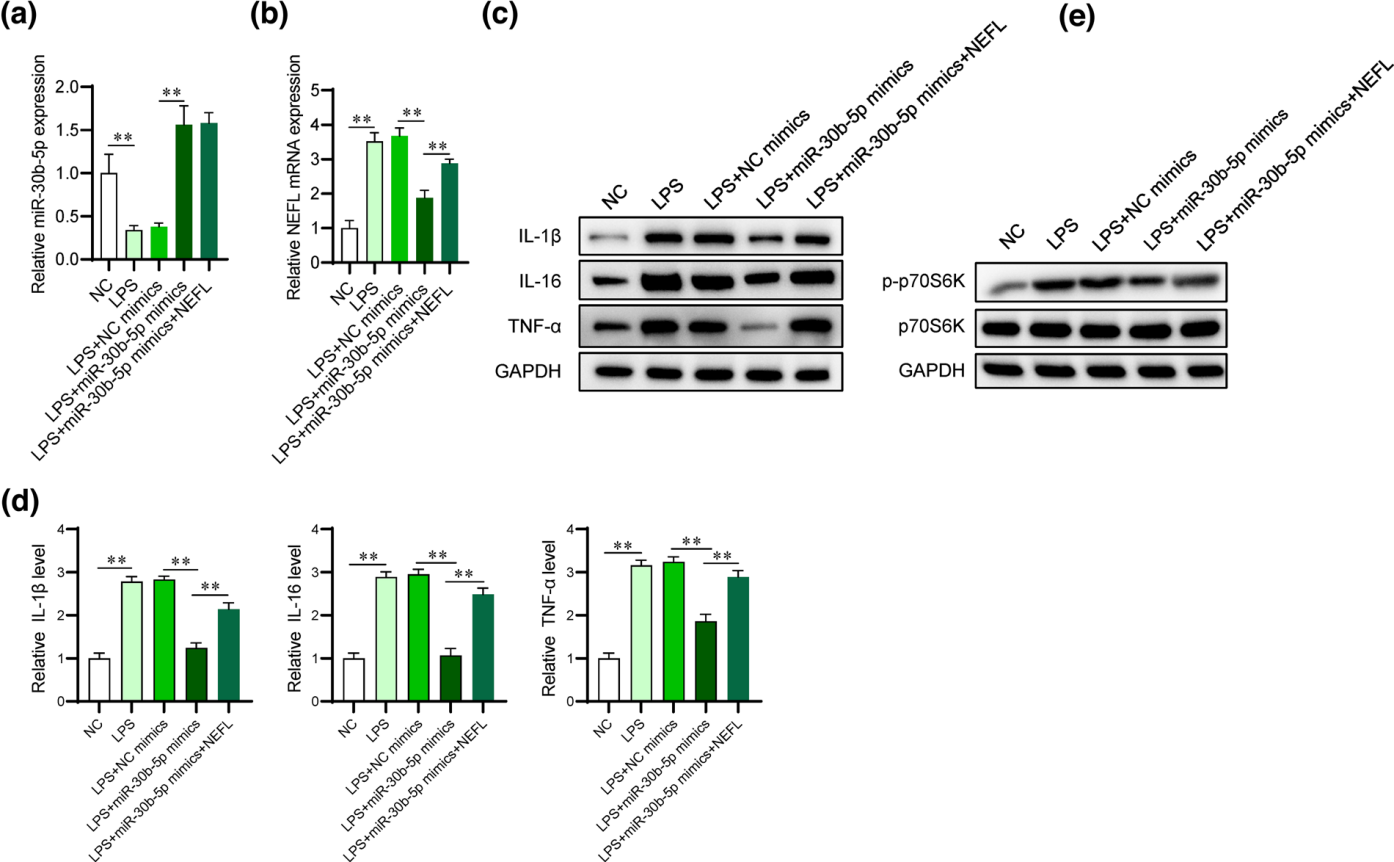
MiR‐30b‐5p inhibits the inflammatory response in LPS‐treated PC12 cells by targeting NEFL. (A) RT‒qPCR analysis was used to detect the levels of miR‐30b‐5p in LPS‐treated PC12 cells after the indicated transfection. (B) The mRNA expression of NEFL in PC12 cells under the indicated treatments was detected using RT‒qPCR analysis. (C‐D) Western blot and RT‒qPCR analyses were performed to assess the protein and mRNA levels of inflammatory cytokines (IL‐1β, IL‐16, and TNF‐α) in PC12 cells under the indicated treatments. (E) Western blotting was used to detect the protein levels of p70S6k and phosphorylated p70S6k in PC12 cells under indicated treatments. **p < 0.01.

The functions and regulatory mechanisms of miR‐30b‐5p were also explored in SCI cell models. The miR‐30b‐5p levels showed a significant decrease in LPS‐stimulated PC12 cells, and the upregulation efficiency of miR‐30b‐5p was confirmed according to RT‒qPCR analysis. The expression of miR‐30b‐5p was not affected after NEFL in LPS‐treated PC12 cells (Figure 6A). Then, we measured the mRNA expression of NEFL in LPS‐treated PC12 cells. The transfection of miR‐30b‐5p mimics reversed the LPS‐induced increase in NEFL expression, which was partially rescued by transfecting NEFL overexpression plasmids (Figure 6B). The LPS‐induced increase in the protein and mRNA levels of IL‐1β, IL‐16, and TNF‐α was also demonstrated to be reduced after miR‐30b‐5p upregulation, and overexpressed NEFL partially offset this effect in LPS‐treated PC12 cells (Figure 6C‐D). NEFL was reported to activate the mTOR signaling pathway in a previous study (Peng et al., 2015). We then detected the levels of phosphorylation of p70S6k, an important downstream substrate of activated mTOR and a known indicator of activated mTOR signaling. As shown in Figure 6E, the p70S6k phosphorylation induced by LPS was inhibited by upregulated miR‐30b‐5p, which was rescued by NEFL upregulation in LPS‐treated PC12 cells.

## 4 DISCUSSION

In this work, an SCI rat model and LPS‐stimulated PC12 cell model were established to elucidate the impact of NEFL on SCI progression in vivo and in vitro. We found that NEFL exhibited significant upregulation in the tissue samples of SCI animal models and PC12 cells under LPS treatment. NEFL silencing attenuated SCI‐induced inflammation and histological changes.

As previously reported, neurofilaments are a main component of the axon cytoskeleton and interact with other cytoskeletal proteins and modulate axonal transportation and neuronal signaling (Yuan et al., 2017). Neurofilaments in neurons are released and increased after neuronal cell damage and are suggested as potential biomarkers for neurological disorders, including SCI (Al‐Chalabi & Miller, 2003; Lee et al., 2020). Neurofilament light chain (NEFL, also named NF‐L) is a subunit of neurofilaments. A previous study reported that NEFL is a marker for neuronal damage and is associated with neuroinflammation (Rosadas et al., 2021). CSF NF‐L expression was significantly increased in amyotrophic lateral sclerosis (ALS) and is of diagnostic and prognostic value for ALS (Rossi et al., 2018). Antibodies to NEFL aggravate neurological disease and may be involved in neurodegeneration progression (Puentes et al., 2017). In the current study, NEFL upregulation was confirmed in SCI rat models and cell models. Knockdown of NEFL attenuated SCI‐induced histological changes in rat spinal tissues and neuronal loss. The increased levels of inflammatory cytokines, including TNF‐α, IL‐1β, and IL‐16, in SCI rat spinal cord tissue samples were significantly reversed after NEFL silencing. The functional recovery of SCI rats was also promoted by NEFL deficiency.

MiR‐30b‐5p was revealed to target NEFL. A previous study revealed that miR‐30b‐5p alleviates neuropathic pain by negatively regulating CYP24A1 to inactivate the Wnt/β‐catenin pathway in chronic constrictive injury rats (Liao et al., 2022). MiR‐30b‐5p is also suggested to exert neuroprotective effects post traumatic brain injury by targeting SEMA3A (Yang et al., 2019). In our study, we found significant downregulation of miR‐30b‐5p in the spinal cord tissue samples of rats after SCI and LPS‐treated PC12 cells. Overexpression of miR‐30b‐5p showed an inhibitory effect on morphological changes in rat spinal cord tissues following SCI and the levels of inflammatory cytokines (IL‐1β, IL‐16 and TNF‐α). Moreover, miR‐30b‐5p upregulation was also demonstrated to promote the functional recovery of SCI rats. The negative regulation of NEFL levels by miR‐30b‐5p was determined, and miR‐30b‐5p attenuated SCI progression by targeting NEFL to inactivate mTOR signaling. The findings of our study were consistent with previous works, and miR‐30b‐5p exerted a neuroprotective effect following SCI.

The mTOR/P70S6K signaling pathway has been demonstrated to be activated by NEFL following SCI. Previous studies have revealed that the mTOR signaling pathway regulates cell growth, proliferation, apoptosis and differentiation, and is critically involved in diseases of the CNS (Kanno et al., 2012). The inhibition of mTOR attenuates the neural tissue damage in brain injuries as well as locomotor impairment following SCI (Huang et al., 2018; Zhang et al., 2021). In our study, we found that the mTOR signaling pathway is suppressed by miR‐30b‐5p overexpression in LPS‐induced PC12 cells, which was rescued after NEFL upregulation. Overexpression of miR‐30b‐5p has also been reported to partially reverse the inhibitory effect of baicalein on the AMPK/mTOR pathway in Parkinson's disease (Chen et al., 2021). The difference from our findings may be explained by the different animal and cell models we used and the influence of other interventions such as baicalein treatments.

However, there were also some limitations in the current study. First, the locomotor function of rats was only evaluated using the BBB scale, which may be influenced by the subjectivity of researchers. Second, the explicit mechanism by which NEFL activates the mTOR signaling was not explored. Third, NEFL overexpression partially reversed the effect of miR‐30b‐5p overexpression on SCI progression, which indicated the existence of other potential mechanisms.

In conclusion, miR‐30b‐5p alleviated the inflammatory response and promoted functional recovery following SCI by targeting NEFL to inactivate mTOR signaling. The findings of our work may expand the knowledge and provide novel insight into SCI‐targeted therapy.

## CONFLICT OF INTEREST

The authors declare that they have no competing interests.

### PEER REVIEW

The peer review history for this article is available at: https://publons.com/publon/10.1002/brb3.2788.

## Data Availability

The datasets during and/or analyzed during the current study are available from the corresponding author on reasonable request.
